# Potentially Avoidable Peripartum Hysterectomies in Denmark: A Population Based Clinical Audit

**DOI:** 10.1371/journal.pone.0161302

**Published:** 2016-08-25

**Authors:** Lotte Berdiin Colmorn, Lone Krebs, Jens Langhoff-Roos, NOSS study group

**Affiliations:** 1 Department of Obstetrics, Rigshospitalet Copenhagen University Hospital, University of Copenhagen, Copenhagen, Denmark; 2 Department of Obstetrics and Gynecology, Holbæk Hospital/University of Copenhagen, Holbæk, Denmark; Duke University, UNITED STATES

## Abstract

**Objective:**

To audit the clinical management preceding peripartum hysterectomy and evaluate if peripartum hysterectomies are potentially avoidable and by which means.

**Material and Methods:**

We developed a structured audit form based on explicit criteria for the minimal mandatory management of the specific types of pregnancy and delivery complications leading to peripartum hysterectomy. We evaluated medical records of the 50 Danish women with peripartum hysterectomy identified in the Nordic Obstetric Surveillance Study 2009–2012 and made short narratives of all cases.

**Results:**

The most frequent indication for hysterectomy was hemorrhage. The two main initial causes were abnormally invasive placenta (26%) and lacerations (26%). Primary atony was third and occurred in 20%. Before hysterectomy another 26% had secondary atony following complications such as lacerations, retained placental tissue or coagulation defects. Of the 50 cases, 24% were assessed to be avoidable and 30% potentially avoidable. Hysterectomy following primary and secondary atony was assessed to be avoidable in 4/10 and 4/13 cases, respectively. Early sufficient suturing of lacerations and uterine ruptures, as well as a more widespread use of intrauterine balloons alone or in combination with uterine compression sutures (the sandwich model), could presumably have prevented about one fourth of the peripartum hysterectomies.

**Conclusion:**

More than 50% of peripartum hysterectomies seem to be avoidable by simple measures. In order to minimize the number of unnecessary peripartum hysterectomies, obstetricians and anesthesiologists should investigate individual cases by structured clinical audit, and disseminate and discuss the results for educational purposes. An international collaboration is warranted to strengthen our recommendations and reveal if they are generally applicable.

## Introduction

Peripartum hysterectomy is a tragedy for a young, fertile woman as it deprives her the chance of having more children. Obstetricians usually consider the hysterectomy as a last option in severe obstetric hemorrhage to save the life of the mother, but the indications differ substantially. Several epidemiological studies of risk factors such as maternal age (≥35years), overweight, high parity, previous cesarean section, mode of delivery and severe hemorrhage have been published [1–4], but in the individual case timely and proper management of severe complications at delivery are essential. Clinical audit is a way to evaluate patient care and outcomes by use of explicit criteria and guidelines and gives the possibility to use clinical assessment of less well defined criteria such as timely and proper management [5].

This paper presents an exploratory structured audit of peripartum hysterectomies based on medical records. This type of research has previously mainly been performed in perinatal and maternal deaths [5–8] and cesarean section [9]. We wanted to evaluate if some peripartum hysterectomies could have been avoided, assessed from a list of explicit criteria for the minimal mandatory medical and surgical interventions preceding hysterectomy together with a clinical evaluation of timely and proper management. A short narrative of all individual cases and the audit evaluation form are available as S1 Table and S1 File.

## Material and Methods

This study is based on results from the Nordic Obstetric Surveillance Study (NOSS) conducted from April 2009 to August 2012, where cases of excessive hemorrhage, uterine rupture, abnormally invasive placenta, and peripartum hysterectomy was reported directly from the clinicians at the participating maternity units. The study period varied slightly between the countries. NOSS included 91% of the Nordic birth cohort during the study period. Peripartum hysterectomies were defined as all emergency hysterectomies performed within 7 days from delivery, excluding hysterectomies due to malignancy. Material, methods, occurrence rates and risk factor analyses has been described elsewhere [1,10–12]. For this specific audit we included all Danish cases of peripartum hysterectomy reported to the NOSS study. The entire Danish birth cohort from 2009–2011 (n = 168 170) with representation of all Danish hospitals (n = 30) was included in NOSS. The number of reported cases was validated by retrieval of cases with ICD-10 (International Classification of Diseases, version 10) and NOMESCO classifications of Surgical Procedures (nowbase.org) regarding hysterectomies, and cases missing in the NOSS database were requested and obtained from the hospitals. A total of 50 cases of peripartum hysterectomy were identified during the study period and all medical records were available and retrieved from the hospitals for the present study. Ethical permission was obtained from the Danish National Board of Health (jrn-Nr 3-3013-681/1/), which also stated that informed written consent from patients was not needed.

The audit committee included the three authors; two senior obstetricians from different hospitals and one young obstetrician. The two senior obstetricians have extensive experience in developing and performing audits in obstetrics and neonatology (5, 7, 8). In a first step, all records were reviewed and possible issues relevant for the management of peripartum hysterectomy were identified. In a second step we wrote narratives, available in S1 Table, and elaborated a structured audit form, available in S1 File, considering the following five dimensions:

1. Indications for peripartum hysterectomy were categorized into 11 groups (Table 1). Atony was divided into primary atony’—the exhausted uterus without other complications—and ‘secondary atony’ subsequent to and explained by other complications such as: the uterus could not contract due to retained placental tissue, coagulation defects, and secondary to excessive blood loss following lacerations. In cases with more obstetric complications preceding hysterectomy we selected the indication as the one obstetric complication initiating the cascade towards hysterectomy.
2. Management of complications preceding hysterectomy was divided into surgical and medical interventions. Timely and appropriate management was defined by explicit criteria for the minimum standard of care inspired by national guidelines (www.DSOG.dk), supplemented by consensus of best clinical practice (Table 2).
3. Postoperative complications following peripartum hysterectomy including re-operation and continued bleeding in spite of the hysterectomy.
4. Maternal characteristic including parity, advanced maternal age and request of sterilization prior to delivery. Women who requested sterilization might have had an increased risk of hysterectomy due to less effort made to avoid hysterectomy.
5. Management of labor was assessed to evaluate if prolonged induction (>24h) or excessive augmentation (>12h) contributed to the risk of hysterectomy?

**Table 1:** Indications for hysterectomy and avoidability.

Indication	*N*	(%)	Avoidable	Potentiallyavoidable	Unavoidable
Laceration	13	(26%)	6	5	2
Accreta*	13	(26%)	0	6	7
Primary atony	10	(20%)	4	2	4
Percreta	3	(6%)	0	0	3
Fibromas	3	(6%)	0	0	3
Complete uterine rupture	3	(6%)	1	1	1
Sepsis	2	(4%)	0	0	2
DIC following HELLP	1	(2%)	0	0	1
Abruptio	1	(2%)	0	1	0
Dehiescence	1	(2%)	1	0	0
Secondary atony**	13	(26%)	4	7	2

* One women with accreta requested hysterectomy.

** Atony in combination with at least one of the above mentioned complications besides primary atony

**Table 2:** Criteria for minimal acceptable medical and surgical intervention.

Complication	Minimal acceptable interventions	
**Atony**	Oxytocin, Prostaglandin, tranexamic acid	Uterine tamponade (tissue/balloon) or compression sutures (e.g. B-lynch). Additional: Sandwich (tamponade AND compression)
**Abruptio**	Oxytocin	Hemostatic sutures (cross stitches etc.)
**Placenta previa**	Oxytocin, tranexamic acid	
**Placenta accreta**	Oxytocin, tranexamic acid	Hemostatic sutures
**Placenta percreta, recognized**	Oxytocin, tranexamic acid	Admittance to referral center, Prophylactic use of Intra vesical balloon catheters, resection of affected myometrium, suturing of lesion and tamponade/compression sutures
**Uterine rupture (including partial rupture)**		Suturing uterus
**HELLP**	Awareness of coagulation parameters, Steroids	Timely delivery
**DIC**	Thrombo-elastography or similar analysis	Balanced transfusions.
**Sepsis**	Relevant antibiotics	Timely diagnosis of sepsis
**Fibromas**	Oxytocin, tranexamic acid	
Laceration and other bleeding*	Tranexamic acid.	Hemostatic sutures. Ligation of arterial vessels. Timely re-suturing

* E.g. lacerations of the genital tract, uterus, intraabdominal bleeding from resection sites, uterine artery and broad ligament

All records were reviewed and the authors assessed if the hysterectomy was 1) avoidable (if suboptimal management was identified that was likely to have influenced the outcome), 2) potentially avoidable (if no predefined suboptimal factors were identified, but the auditors found that the hysterectomy possibly could have been avoided with additional measures), 3) unavoidable (if no suboptimal factors were identified and all recommended interventions were made). The reviewers were not blinded to each other.

## Results

Maternal characteristics of the 50 women with peripartum hysterectomy and the background population are shown in Table 3: The groups did not differ in relation to maternal age, BMI, parity, rate of twin pregnancies and labor induction. Women with peripartum hysterectomy were more often multi-parous (aOR 2.8), with a history of previous cesarean section (aOR 3.7), and more often delivered preterm (aOR 3.7) by emergency or planned cesarean delivery (aOR 8.1 and 4.2 respectively). Information of blood loss, transfusions, sterilization and complications to hysterectomy are shown in Table 4.

**Table 3:** Maternal characteristics and risk factors.

	Hysterectomy(n = 50)	Background population^a^ (n = 168 187)	aOR (95%CI)^b^	*P-value*^b^
	N (%)	N (%)		
**Age**				*0*.*592*
**<35 years**	30 (60%)	127 889 (76%)	1	
**35 years or more**	20 (40%)	40 298 (24%)	1.2 (0.65–2.15)	
**BMI**				*0*.*559*
**<25**	30 (60%)	99 111 (59%)	1	
**25–29**	12 (24%)	38 367 (23%)	0.77 (0.39–1.51)	
**30 or more**	8 (16%)	21 992 (13%)	0.69 (0.31–1.5)	
**Previous deliveries**				*0*.*046*
**0**	12 (24%)	74 392 (44%)	0.7 (0.31–1.62)	
**1**	20 (40%)	60 938 (36%)	1	
**2**	10 (20%)	22 156 (13%)	1.39 (0.64–3.01)	
**3 or more**	8 (16%)	7 598 (5%)	2.84 (1.21–6.68)	
**Previous CS**	28 (56%)	19 404 (12%)	3.38 (1.63–7.01)	*0*.*001*
**Twins or more**	3 (6%)	3281 (2%)	0.65 (0.19–2.24)	*0*.*496*
**GA (full weeks)**				*0*.*0004*
**Preterm <37weeks**	15 (30%)	9684 (6%)	3.74 (1.9–7.4)	
**Term 37-41weeks**	34 (68%)	148 146 (88%)	1	
**Post term 42 or more**	1 (2%)	8665 (5%)	0.46 (0.06–3.45)	
**Labor induction**	10 (20%)	29 841 (18%)	1.52 (0.72–3.23)	*0*.*274*
**Mode of delivery**				*<0*.*0001*
**Vaginal**	12 (14%)	132 378 (79%)	1	
**Emergency CS**	24 (48%)	19 168 (11%)	8.06 (3.69–17.57)	
**Planned CS**	14 (28%)	16 653 (10%)	4.17 (1.71–10.16)	

^a^ missing data not shown.

^b^ aOR’s and p-values are calculated by multiple logistic regression and adjusted for all shown variables

**Table 4:** Obstetric complications related to peripartum hysterectomy.

	N (%)
**Estimated blood loss**	
Less than 1000 ml	4 (8%)
1000–4999 ml	10 (20%)
5000–9999 ml	19 (38%)
10000 ml or more	18 (36%)
**Transfusion with RBC (units)**	
0–5	11 (22%)
6 or more	39 (78%)
**Medical complications after hysterectomy**	11 (22%)
**Maternal death**	1 (2%)
**Reoperation after hysterectomy**	6 (12%)

The complications initiating the cascade or train of events that eventually led to hysterectomy are listed in Table 1. Overall, 47 of the hysterectomies were related to hemorrhage, two to sepsis and one was upon maternal request due to severe pregnancy complications and placenta accreta. The leading causes of hemorrhage were abnormally invasive placenta (26%) or lacerations (26%). Uterine atony complicated almost half of all cases (46%), however in 26% atony were secondary to other complications, mostly abnormally invasive placenta or lacerations. Only 20% were primary atony without other complications (Table 1). Almost three quarters of the women had more than one complication preceding hysterectomy, most often complications followed by secondary atony. In women with abnormally invasive placenta, hysterectomy was mostly performed due to bleeding after incomplete removal of an accrete or increte placenta or impossible resection of a percrete placenta. Lacerations were in all but one case iatrogenic and occurred during cesarean section, often followed by secondary atony. Short narratives and indications for hysterectomy of all cases are given in S1 Table.

Six women (12%) were re-operated after the hysterectomy. One woman developed Ogilvie’s syndrome (colonic pseudo-obstruction with massive dilatation of the cecum and right colon). In spite of disinflation, she still had a perforated colon and subsequently a hemicolectomy. The other five women were re-operated due to intra-abdominal bleeding from the resection sites from the hysterectomy. A total of 11 women developed complications following the hysterectomy. Two women developed cardiac arrest during surgery. One hysterectomy was performed on vital indication after 3500 ml of bleeding. The woman died despite trial of resuscitation. The other woman developed heavy postpartum bleeding due to primary atony and bled 6000 ml within a short time (1 hour). She had cardiac arrest during hysterectomy and was resuscitated. Postoperatively she developed hemolysis and adult respiratory distress syndrome. Other major complications were pulmonic edema, pulmonary micro-embolism, transient kidney insufficiency, acute tubular necrosis, transient Sheehan’s syndrome, ischemia of the optic nerve with partial loss of sight, peripheral neuropathy and thrombotic thrombocytopenic purpura.

Women with request for sterilization, higher parity (≥2 previous deliveries) or higher maternal age (≥35 years) were not overrepresented in the groups with avoidable or potentially avoidable hysterectomies compared to those with unavoidable hysterectomies (Table 5).

**Table 5:** Sterilization, parity and maternal age as predictors for avoidability.

	Unavoidable N = 24		Potentially avoidable N = 14		Avoidable N = 12		P-value*
	n	%	n	%	n	%	
**No sterilization**	18	75%	10	71%	12	100%	*p = 0*.*168*
**Sterilization**	6	25%	4	29%	0	0%	
**Less than 2 previous deliveries**	12	50%	10	71%	10	83%	*p = 0*.*115*
**2 or more previous deliveries**	12	50%	4	29%	2	17%	
**Age less than 35y**	11	54%	10	71%	10	83%	*p = 0*.*064*
**Age 35y or more**	13	46%	4	29%	2	17%	

*p-values are calculated by chi-square or Fischer’s exact test when needed

According to the criteria for minimal management we found that 12 (24%) of the hysterectomies were avoidable due to suboptimal interventions and management of complications leading to hysterectomy. In 15 women (30%) we identified no direct suboptimal factors, but the hysterectomy could possibly have been avoided if additional intervention had been attempted. For 23 women (46%) we identified no suboptimal factors or possibility for additional intervention and the hysterectomy was assessed as being unavoidable (Table 1). For avoidable hysterectomies the most common suboptimal factors identified were insufficient or untimely suturing of lacerations or uterine rupture (complete and dehiscence) leading to excessive hemorrhage or secondary atony and hysterectomy (n = 8, 66% of all avoidable cases). In three of these avoidable cases with lacerations, the uterine artery was involved, but an attempt to ligate the artery was not described. In the avoidable cases with primary atony (n = 4), the most common suboptimal factors were absence of uterine tamponade (balloon or likewise) or compression sutures (B-lynch or likewise). In one case a balloon was used, but with less than the recommended amount (500 ml) of water for compression. In one case, delay of intervention through the entire delivery was thought to be a major suboptimal factor leading to hysterectomy, and in another case the minimal recommended medical treatment was not given.

In 15 cases, a different management of labor or attempt of additional interventions, not part of a minimal expected intervention, could possibly have prevented the hysterectomy. In cases with placenta accreta, primary atony, placental abruption and laceration with secondary atony a more frequent use of intra-uterine balloon as tamponade either alone, or in combination with uterine compression sutures, to make a compression ‘sandwich’ could presumably have prevented some hysterectomies. In a woman with previous cesarean section, prolonged induction was associated with complete rupture resulting in subsequent hysterectomy.

## Discussion

By evaluating the Danish cases we found that almost all women in the reported cases received the minimal recommended medical intervention before the hysterectomy. Meanwhile, less extensive surgical interventions were not always attempted. Of the 50 cases, 24% were assessed to be avoidable and 30% potentially avoidable. Due to the small number of cases these percentages have large confidence limits. We nevertheless conclude from our sample that some hysterectomies could have been avoided by; 1) early and sufficient sutures of lacerations and incomplete partial and total uterine ruptures, 2) correct use of intrauterine balloons in case of hemorrhage, or 3) the sandwich method—a combination of balloon and uterine sutures [13].

Danish women with peripartum hysterectomy were more often multi-parous, with previous cesarean section and more often delivered preterm and by cesarean section; otherwise they did not differ substantially from the background population. These findings are consistent with the results from previous studies [1,2,4,12], although some studies also found that higher maternal age and labor induction were predictors of peripartum hysterectomy.

As expected, most peripartum hysterectomies were associated with excessive hemorrhage (94%). Overall, uterine atony (46%) was the most frequent cause of hemorrhage, but designating the cause initiating the cascade to hysterectomy, lacerations (26%) and abnormally invasive placenta (26%) were more common. Only in 20% of the cases, uterine atony was described as primary uterine atony—the initiating cause.

The cause of hysterectomy can be one or more clinical complications. In this study we chose to consider causes as primary when they were first in time in the train of events. The hierarchy of causes is not always included in clinical reports, and in the literature opinions of what are the most important causes and indications differ. Causes can be primary in time or the last in the train of event leading to hysterectomy or they can be the most serious from a clinical point of view. In the literature the selection is not always clear. All but one [2] of the reviewed studies [2,3,10–12,14] support the finding of abnormally invasive placenta as a very frequent cause of hemorrhage leading to hysterectomy, involved in about 30% to 50% of all cases. Two of the studies [10,14] designated uterine atony as the most frequent cause of hemorrhage (53% and 37% respectively) leading to peripartum hysterectomy without distinction between primary and secondary uterine atony. One study [3] summoned all hemorrhage not related to placenta previa, accreta or placental abruption in one category causing 37% of all peripartum hysterectomies. Only one study [12] clearly distinguished between primary uterine atony (27%) and uterine atony in combination with other complications (6.3%). Lacerations were described in four studies [10–12,14] as a cause of hemorrhage and hysterectomy in the range, 4–41% but with different definitions. Most studies [2,3,10,14] reported all identified causes involved in hemorrhage, while two studies [11,12] reported only the most important indication for hysterectomy. Uniform classification and clear definitions of causes and indications for hemorrhage and hysterectomy are important in the evaluation of obstetric care to enable cross-country studies. Also it is important to differentiate between causes (clinical complications) and disposing risk factors (age, BMI, obstetric history), which in some publications are pooled as independent variables in regression analyses.

Epidemiological studies and randomized controlled trials provide insights regarding incidences, risk factors, management and outcomes, while case reviews and audits add qualitative information about the standard of care to understand the questions of ‘why’ and ‘how to improve’. The differences between epidemiology and audit imply several methodological challenges due to divergent opinions of optimal care depending on experts, nations, locations and over time [5,15]. Not all maternity units have the same facilities or tradition for management of obstetric emergencies. Consensus of a minimal mandatory management must therefore consist of interventions expected to be accessible in all delivery units in the studied population. The management should be based on nationally accepted guidelines if available, or if missing, consensus of best practice. For this audit we developed a set of explicit criteria for the minimal acceptable medical and surgical intervention based on National guidelines and consensus of best clinical practice (www.DSOG.dk). The sandwich method was included as part of the minimal acceptable intervention in relation to hemorrhage, although not presently described in the Danish national guidelines. The sandwich model has been suggested as a method for second line therapy in cases of severe postpartum hemorrhage in several studies and represents a more progressive management to avoid peripartum hysterectomies [13,16]. Compression is probably the most important measure to limit and stop the hemorrhage. However, case notes do not contain information on compression techniques in detail. The description of methods, duration or efficacy of compression would be very fragmented and insufficient. We therefore did not include compression techniques such as aorta compression in our criteria for minimal mandatory management.

Case reviews can draw attention to the clinical challenges of rare and severe complications in pregnancy and childbirth. The acquired knowledge can improve the clinical insight of young or less experienced obstetricians and motivate implementation of new recommendations in National guidelines. All information must be available for the evaluation to be valid, when reviewing cases for audits. Hence, the source of information has to be carefully evaluated. Existing databases and registers are well known ways to identify cases and access their data, although valuable information could be lost due to insufficient or incomplete registration. Medical records are considered as gold standard and provide the best source of information, but even those are in some cases incomplete.

The necessary size and composition of the audit committee to give an objective evaluation of the cases was discussed. Evaluation from a small group could be biased by narrow-minded views on what is appropriate obstetric care, whereas a larger group could have difficulties in obtaining agreement and be more inclined to compromise on a less progressive view. We decided to keep the group small with representatives of more and less experienced obstetricians from both highly and less specialized units. Moving to an international setup, the committee should include experts from all specialties involved to ascertain a more nuanced assessment.

We believe clinicians do their best to prevent obstetric complications at delivery. Peripartum hysterectomy is regarded as the last option available, when interventions to save the uterus would endanger the woman’s health and potentially be life threatening compared to a hysterectomy. In general it is essential to keep in mind that peripartum hysterectomy may be a lifesaving procedure; the caretaker should always consider prevention of mortality first, even if this requires the morbidity of an unplanned hysterectomy. Deciding on life saving procedures relies on complex assessments in acute situations. The clinician is often under extensive pressure and the management depends on available interventions, hospital facilities, obstetric experience, surgical skills and subjective considerations of maternal characteristics. In Denmark where obstetricians and gynecologist may share the night duty, a gynecologist may be more prone to perform a familiar procedure, such as a hysterectomy, instead of less known obstetric procedures where simple sutures are more often used. The impact of the surgeon’s subspecialty was not assessed in this audit. The importance of this aspect is discussed in a study from Australia where the maternal morbidity was shown to depend on which surgeons were present at the surgery [17].

We have developed a simple assessment form for the management of complications preceding peripartum hysterectomy and drawn attention to updated strategies to improve the future critical, obstetric care in Denmark. To optimize the evidence for clinical guidelines, we recommend that audits of rare and severe complications—like peripartum hysterectomy—should be performed in an international setting like the International Network of Obstetric Survey Systems (INOSS, www.npeu.ox.ac.uk/inoss). INOSS is a collaboration for obstetric surveillance systems and conduct studies of rare and severe obstetric complications by use of unified definitions, methodology and specifically collected data [18]. International case reviews would be challenged by diverse traditions in medical record keeping and accessibility, language difficulties, diverse definitions, coding systems and coding practices, and differences in facilities even among high-income countries. The criteria for minimal mandatory treatment used in this audit may be applicable as minimal mandatory interventions in most maternity units in Europe, and colleagues should discuss the criteria in an international setting for educational purposes.

## Supporting Information

S1 Table Short narratives. (DOCX)

S1 File Audit evaluation form for peripartum hysterectomy. (DOCX)
