# Supplementary material for: Potentially Avoidable Peripartum Hysterectomies in Denmark: A Population Based Clinical Audit
Source: PLoS One. 2016 Aug 25;11(8):e0161302. doi: 10.1371/journal.pone.0161302 (PMC4999193; doi:10.1371/journal.pone.0161302)
Supplement: S1 Table — (DOCX) [file pone.0161302.s001.docx]

**Supporting Information**

**S1 Table. Short narratives.**

| ID | Comment | Indication | Blood loss (ml) | Avoidable? |
| --- | --- | --- | --- | --- |
| 1 | 29 years, para 1, 38 weeks. Instrumental vaginal delivery with primary atoni following long induction (17h) and hyperstimulation. Sufficient medical and surgical interventions, but no attempt to combine B-lynch sutures and balloon. | Primary atony | 10 000 | Potentially |
| 2 | 33 years, para 1, 41 weeks. One previous CS. Emergency CS due to suspicion of uterine rupture and FHR changes. Laceration of uterine artery; insufficiently sutured. Ligation not tried. Dehiescence sutured. Rapid decision to perform hysterectomy. | Laceration and secondary atony | 1 800 | Yes |
| 3 | 32 years, para 2, 41 weeks. Instrumental vaginal delivery. Accreta with manual removal and secondary atony. No intrauterine tamponade; a sandwich could have been tried. | Accreta | 12 500 | Potentially |
| 4 | 26 years, para 0, 40 weeks. Emergency CS due to prolonged expulsion. Laceration including the uterine artery. Insufficient ligation and suturing leading to secundary atony with hysterectomy at second reoperation. No use of compression or tamponade. | Laceration and secondary atony | 10 000 | Yes |
| 5 | 35 years, para 3, 33 weeks. Preterm emergency CS due to bleeding from praevia and asphyxia. Accrete placenta with continuous vaginal bleeding despite tamponade. Hysterectomy at re-operation. Request of sterilization. | Accreta | 11 000 | No |
| 6 | 33 years, para 0, 41 weeks. Induction of labour. Active phase 17h induction, fully dilated for 3 h, pushing 75 min, retained placenta for 1h. Primary atoni with DIC. No surgical intervention (compression sutures or balloon). | Primary atony | 10 300 | Yes |
| 7 | 35 years, para 5, 36 weeks. Planned CS due to praevia and accreta. Bleeding from accreta and laceration leading to hysterectomy. Sufficient medical and surgical intervention. Request of sterilization. Re-operated due to bleeding from surgical sites. | Accreta and Laceration | 13 000 | No |
| 8 | 25 years, para 1, 38 weeks. Bicorn uterus. One previous CS. Planned CS on maternal request. Post operative primary atony. Two re-operations, the first with DIC. Hysterectomy at second re-operation. Sandwich could have been applied at first reoperation. | Primary atony | 11 000 | Potentially |
| 9 | 43 years, para 6, 36 weeks. Preterm emergency CS due to IUGR, asphyxia and preeclampsia. Lacerations due to adherences, followed by DIC and secondary atony. Succifient intervention. Reoperation with hysterectomy due to intra-abdominal bleeding. Complicated postoperative with HELLP. Request of sterilization. | Lacerations and secondary atony with DIC | 8 500 | No |
| 10 | 46 years, para 0, 31 weeks. Initial request for abortion. Pregnant after transcervical endometrial resection (TCER). Preterm emergency CS due to IUGR and asphyxia. Accrete placenta was suspected due to TCER. The woman wanted hysterectomy if the surgeon thought conservative treatment would imply any risk of complications. | Accreta/increta | 1 000 | No |
| 11 | 32 years, para 0, 41 weeks. Emergency CS due to slow progression of labor, followed by postoperative atony. Insufficient medical intervention and less than 500ml water in the balloon. Hysterectomy at re-operation. | Primary atony | 4 800 | Yes |
| 12 | 34 years, para 1, 39 weeks. Instrumental vaginal delivery, shoulder dystocia, insufficient uterine contraction. Postpartum suspicion of rupture due to abdominal pain and intra-abdominal fluid. Delay of and insufficient intervention at laparotomy with hysterectomy. No attempt to resuture. | Complete rupture (post partum) | 6 300 | Yes |
| 13 | 38 years, para 1, 38 weeks. Labor induction (Twins). Emergency CS due to slow progression. Accreta and secondary atony. Sufficient medical intervention and B-lynch. Could have tried sandwich (No balloon) | Accreta and secondary atony | 10 000 | Potentially |
| 14 | 33 years, para 1, 40 weeks. Emergency CS due to slow progression and suspicion of uterine rupture. Accreta/Increta and bleeding. No use of balloon or compression sutures. | Accreta | 6 000 | Potentially |
| 15 | 30 years, para 1, 41 weeks. Very fast vaginal delivery with post-partum primary atony and cervical laceration. Sufficient medical and surgical intervention Laparotomy with hysterectomy due to heavy bleeding. | Primary atony | 7 500 | Yes |
| 16 | 40 years, para 3, 38 weeks. Induction (55h) due to cholestasis. Vaginal delivery. Placental retention and atony. Sufficient medical and surgical intervention. Laparotomy with hysterectomy. Cardiac arrest with resucitation during hysterectomy. Re-operation due to bleeding from surgical sites. No sequelae. | Primary Atony | 8 800 | No |
| 17 | 34 years, para 0, 41 weeks. Instrumental vaginal delivery due to loss of consciousness during pushing. Primary atony with heavy vaginal bleeding, sufficient medical intervention. DIC. Laparotomy with hysterectomy on vital indication. No time for additional surgical intervention. Complicated post-operative course. | Primary atony (amniotic embolism?) | 14 000 | No |
| 18 | 47 years, para 2, 38 weeks. Planned CS due to praevia. Request for sterilization. First re-operation due to secondary atony and bleeding from salpinges. Sufficient intervention. Second re-operation with hysterectomy due to continued circulatory instability. Could have used balloon already after CS with praevia and a bleeding of 1L. | Laceration and secondary atony | 8 000 | Potentially |
| 19 | 35 years, para 1, 38 weeks. One previous CS.Emergency CS due to suspicion of uterine rupture. Postoperative secondary atony. Sufficient medical and surgical intervention. Postoperative complicated with O’gilvie’s syndrome, perforation of the colon and hemicolectomy. | Complete rupture and secondary atony | 8 500 | No |
| 20 | 34 years, para 0, 33 weeks. Planned preterm CS due to IUGR. Placenta praevia and accreta followed by secundary atony. Sufficient intervention. Reoperated with hysterectomy due to continuous uncontrollable bleeding from placental bed ?. | Praevia, accreta and secondary atony | 8 000 | No |
| 21 | 30 years, para 1, 41 weeks. Spontaneous vaginal delivery. Hysterectomi six days postpartum due to sepsis from endometritis with group A haemolytic strepstococcus. Relevant antibiotic treatment. | Sepsis | 1 250 | No |
| 22 | 31 years, para 1, 38 weeks. One previous CS. Emergency CS on maternal request. Postoperative atony. Sufficient medical intervention. Reoperation with hysterectomy and no additional surgical intervention (neither compression sutures nor intrauterine tamponade). | Primary atony | 7 500 | Yes |
| 23 | 31 years, para 0, GA 37 weeks. Gemelli. Intended vaginal delivery with labor induction. Emergency CS due to slow progression. Heavy bleeding from lacerations (2L). Post operative Secondary atony with laparotomy and hysterectomy. Maybe lacerations was insufficient sutured at CS. Sufficient interventions at reoperation. | Lacerations and secondary atony | 9 000 | Potentially |
| 24 | 39 years, para 1, 34 weeks. One previous CS. Planned CS due to percreta. Attempt of resection, but too much bleeding. Sufficient medical and surgical intervention. | Percreta | 16 000 | No |
| 25 | 36 years, para 2, 34 weeks. Emergency CS due to suspicion of uterine rupture. It was placental abruption and no rupture. B-lynch applied, but a sandwich could have been tried. Secondary atony and reoperation with hysterectomy. Uterine tamponade should have been tried. Request of sterilization. | Abruptio and secondary atony | 10 000 | Potentially |
| 26 | 35 years, para 1, 40 weeks. Emergency CS due to suspicion of rupture. Lacerations insuccificiently sutured, leading to two reoperations due to bleeding from uterine artery and hysterectomy at second reoperation. | Laceration | 7 600 | Yes |
| 27 | 33 years, para 1, 40 weeks. Vaginal delivery with postpartum endometritis and sepsis with group A streptococcae. Hysterectomy on vital indication. | Sepsis | 1 500 | No |
| 28 | 35 years, para 0, 40 weeks. Emergency CS due to failure of progression after labor induction. Complicated due to severe endometriosis and lacerations. Sufficient medical and surgical intervention. | Laceration | 12 000 | No |
| 29 | 32 years, para 1, 39 weeks. Emergency CS due to slow progression of labor; lacerations insufficiently sutured. Reoperation with hysterectomy due to secondary atony. B-lynch applied but could have tried Sandwich. | Laceration and secondary atony | 7 000 | Potentially |
| 30 | 30 years, para 2, 33 weeks. Twins. Emergency CS due to HELLP. Complicated by DIC and severe uncontrollable hemorrhage. Request of sterilization. | DIC following HELLP | 19 000 | No |
| 31 | 40 years, para 0, 38 weeks. Planned CS due to multiple fibromas; difficult to suture. B-lynch not possible due to adhesions and fibromas. | Fibromas | 5 000 | No |
| 32 | 30 years, para 2, 35 weeks. Emergency CS due to pre-eclampsia. Findings of dehiescence and secondary atony. No attempt to re-suture dehiescence. | Dehiescence and secondary atony | 950 | Yes |
| 33 | 28 years, para 5, 33 weeks. Emergency CS due to antepartum hemorrhage from known praevia and percreta. Not possible to resect placenta. Request of sterilization. | Percreta | 4 500 | No |
| 34 | 38 years, para 1, 38 weeks. Planned CS with placenta accreta and bleeding. Postoperative secondary atony and reoperation with hysterectomy. Second reoperation due to continued intra-abdominal bleeding. | Accreta and secondary atony | 11 500 | Potentially |
| 35 | 34 years, para 0, 42 weeks. Emergency CS after attempt of instrumental vaginal delivery. Lacerations insufficiently sutured. Postoperative secondary atony and reoperation with hysterectomy. Extent of the laceration into the vagina was first recognized after hysterectomy. | Laceration and secondary atony | 10 350 | Yes |
| 36 | 40 years, para 0, 35 weeks. Emergency CS due to pre-eclampsia and multiple fibromas. Not possible to acchieve sufficient hemostasis due to fibromas. | Fibromas | 4 000 | No |
| 37 | 40 years, para 1, 30 weeks. Emergency CS after complicated pregnancy with placenta praevia, accreta and PPROM. Hysterectomy on maternal request due to pregnancy complications. | Praevia accreta, maternal request | 500 | No |
| 38 | 36 years, para 1, 41 weeks. One previous CS. Emergency CS due to suspicion of uterine rupture. Lacerations insufficiently sutured; post operative intraabdominal bleeding and reoperation with hysterectomy. | Laceration | 14 000 | Yes |
| 39 | 32 years, para 2, 35 weeks. Emergency CS due to vaginal hemorrhage from placenta praevia. Increte placenta. Sufficient intervention | Accreta/increta | 4 500 | No |
| 40 | 19 years, para 0, 40 weeks. Vaginal delivery with primary atony and heavy bleeding; Cardiac arrest during intervention and hysterectomy on vital indication; maternal death. | Primary atony | 6 000 | No |
| 41 | 35 years, para 2, 38 weeks. Vaginal delivery with continuous postpartum vaginal bleeding not responding to medical treatment. Intrauterine palpation finds large inresectable fibromas. Hysterectomy due to continuous bleeding. Sufficient intervention. | Fibroma and secondary atony | 9 000 | No |
| 42 | 34 years, para 2, 32 weeks. Planned CS due to placenta percreta. Resection not possible. Request of sterilization. | Percreta | 7 000 | No |
| 43 | 34 years, para 5, 37 weeks. Planned CS due to multiple previous CS, placenta praevia and increta. Resection of placenta not possible. | Accrete/increta | 25 000 | No |
| 44 | 34 years, para 1, 37 weeks. Planned CS due to praevia. Placenta accrete. Haemostatic sutures and intrauterine tamponade applied but a sandwich could have been made. Request of sterilization. | Accreta | 5 200 | Potentially |
| 45 | 34 years, para 3, 41 weeks. Emergency CS due to dysproportion with lacerations and haematoma. Insufficient attempt of resuturing lacerations before hysterectomy. | Laceration | 1 600 | Yes |
| 46 | 27 years, para 1, 40 weeks. Emergency CS due to vaginal bleeding in labour; findings of dehiscence and lacerations. Reoperation due to postoperative vaginal bleeding; and bleeding from the uterotomy. Second reoperation with hysterectomy, due to continued intra-abdominal bleeding . Possibly insufficient suturing of dehiescence and lacerations. | Laceration and dehiescence | 6 600 | Potentially |
| 47 | 37 years, para 2, 38 weeks. Two previous CS. Planned CS with bleeding from lower segment. Sufficient treatment including sandwich without effect. | Primary atony | 5 900 | No |
| 48 | 42 years, para 3, 32. Emergency CS due to PPROM and suspicion of amniotis,. Placenta accreta/increta due to TCER: not possible to remove. Resection not tried. The woman had accepted hysterectomy if increased risk of complications at surgery. Request of sterilization. | Accrete/increta | 1 000 | No (possibly in a unit specialized in resection of placenta percreta) |
| 49 | 28 years, para 2, 40 weeks. No previous CS. Emergency CS after prolonged induction of labour, asphyxia and bradycadia. Known bicorn uterus. Findings of complete major rupture, uterus detached from vagina, impossible to resuture. | Complete uterine rupture | 600 | Potentially |
| 50 | 28 years, para 1, 40 weeks. Vaginal delivery with vaginal laceration treated with suturing and vaginal tamponade. Secondary atony with laparotomy and hysterectomy. Possibly the vaginal tamponade was removed to soon and it could have been supplied with intrauterine tamponade (balloon) for double compression. | Vaginal laceration and secondary atony | 14 000 | Potentially |
