# Supplementary material for: Potentially Avoidable Peripartum Hysterectomies in Denmark: A Population Based Clinical Audit
Source: PLoS One. 2016 Aug 25;11(8):e0161302. doi: 10.1371/journal.pone.0161302 (PMC4999193; doi:10.1371/journal.pone.0161302)
Supplement: S1 File — (DOCX) [file pone.0161302.s002.docx]

# Supporting Information.

# S1 File. Audit evaluation form for peripartum hysterectomy

**Patient ID _________________**

**Maternal age (year) __________________**

**Parity (N, >22GA) ____________________**

**Previous CS (no)_________________**

**Request for sterilization:**

**yes________ no________ unknown__________**

**Informed consent of hysterectomy in case of complications:**

**yes________ no________ unknown__________**

1. **Complications preceding hysterectomy:**

|  | **Type** | **Initial (in order of appearance)** | **Eventual/final/decisive**  **(the complication that was decisive for hysterectomy)** |
| --- | --- | --- | --- |
| *1* | *Atony* |  |  |
| *2* | *Abruptio* |  |  |
| *3* | *Placenta praevia* |  |  |
| *4* | *Placenta Accreta* |  |  |
| *5* | *Placenta percreta, recognized* |  |  |
| *6* | *Uterine rupture (incl incomplete rupture)* |  |  |
| *7* | *HELLP* |  |  |
| *8* | *DIC* |  |  |
| *9* | *Sepsis* |  |  |
| *10* | *Fibromas* |  |  |
| *11* | *Laceration* |  |  |
| *12* | *Other bleeding* |  |  |
| *13* | *Other indications (specify):*  *_______________________________* |  |  |

1. **Management of complications preceding hysterectomy:**

*Did the patient perceive the minimal acceptable medical and/or surgical intervention related to the involved complications?*

| Complication | Min medical interventions | note | Min surgical interventions | note | transfusion | note | Acceptable  Intervention? |
| --- | --- | --- | --- | --- | --- | --- | --- |
| *Atony:* | Oxytocin, Prostglandin, tranexamic acid |  | Uterine tamponade (tissue/balloon) or compression sutures (eg Blynch). Sandwich (tamponade AND compression)? |  |  |  |  |
| *Abruptio* | Oxytocin |  |  |  |  |  |  |
| *Placenta praevia* | Oxytocin, tranexamic acid |  | Hemostatic sutures (cross stitches etc) |  | Blood available |  |  |
| *Placenta Accreta* | Oxytocin, tranexamic acid |  | Hemostatic sutures |  | Blood available |  |  |
| *Placenta percreta, recognised* | Oxytocin, tranexamic acid |  | Intravesical balloons, resection, suturing of lesion and tamponade/compression sutures |  | Blood available |  |  |
| *Uterine rupture (incl incomplete rupture)* |  |  | Re-suturing uterus |  |  |  |  |
| *HELLP* | Awareness of coagulation parameters. |  | Steroids? Timely delivery |  |  |  |  |
| *DIC* | TEC or similar analysis |  | Balanced transfusions |  | According to TEC |  |  |
| *Sepsis* | Relevant antibiotics |  | Timely diagnosis of sepsis |  |  |  |  |
| *Fibromas* | Oxytocin, tranexamic acid |  |  |  |  |  |  |
| *Laceration* | Tranexamic acid |  | Timely re-suturing |  |  |  |  |
| *Other bleeding* | Tranexamic acid |  | Hemostatic sutures  Ligation |  |  |  |  |
| *Other complications (specify):* | resucitation |  |  |  |  |  |  |

1. **Were there any postoperative complications?**
   1. *Did the woman have any complications due to the peripartum hysterectomy?*
      1. *Re-operations: indication, number, time from hyst*
      2. *Organ damage, specify:*
      3. *Medical complications (eg thrombosis, o’gilvie etc), specify:*
   2. *If the hysterectomy was associated with hemorrhage, did the hysterectomy then stop the bleeding? Yes/No*
2. **Management of labor.**
   1. *Did the woman experience prolonged induction of labor(>24hours)?*

*Yes_________ No_________ Unknown___________*

- 1. *Did the woman experience excessive augmentation(>12hours)?*

*Yes_________ No_________ Unknown___________*

- 1. *Were any known risk factors (eg previous CS) for labor complications neglected or treated in discrepancy with existing guidelines?*

*Yes_________ No_________ Unknown___________*

*If Yes, specify___________________________________________________________________________________________________________________________________________________________________________________________________________________________________________________________________________________________________________________________________________________________________________________________*

1. **Evaluation.**
   1. *Were any suboptimal factors identified?*

*Yes_________ No_________ Unknown___________*

- 1. *If yes, were they likely to have influenced the risk for hysterectomy?*

*Yes_________ No_________ Unknown___________*

- 1. *Was the hysterectomy potentially avoidable?*

*Yes_________ No_________ Unknown___________*

1. **Comments.**
